# Examining Long-Term Mental Health Outcomes Associated with Childhood Gun Violence Exposure: Variations by Race/Ethnicity and Gender

**DOI:** 10.1007/s11524-025-01034-2

**Published:** 2025-12-06

**Authors:** Sicong Sun, Darrell L. Hudson, Hedwig Lee

**Affiliations:** 1 Department of Social Welfare, Luskin School of Public Affairs, University of California, 337 Charles E. Young Drive East, Los Angeles, CA 90095 USA; 2 Department of Health Behavior and Health Equity, School of Public Health, University of Michigan, 1415 Washington Heights, Ann Arbor, MI 48109 USA; 3 Department of Sociology, Trinity College of Arts & Sciences, Duke University, 417 Chapel Dr, Durham, NC 27708 USA

**Keywords:** Firearm, Community violence, Depression, Cigarettes, Heavy episodic drinking

## Abstract

This study examined the long-term mental health outcomes associated with childhood gun violence exposure by race/ethnicity and gender. Data were drawn from the National Longitudinal Survey of Youth 1997 (1997–2021). Gun violence exposure was measured as any exposure before age 18. Mental health outcomes assessed included depressive symptoms, heavy episodic drinking, and daily cigarette smoking. Hierarchical generalized linear models were used. Findings indicate that about 16.15% of the respondents reported childhood exposure to gun violence before the age of 18. Gun violence exposure was significantly associated with depressive symptoms among white men, white women, and Hispanic women. Additionally, it was associated with higher odds of heavy episodic drinking among Black men, white women, and Hispanic women. Gun violence exposure was associated with higher odds of daily smoking for all groups. The study findings suggest that there is a high prevalence of childhood exposure to gun violence in the U.S. The relationship between childhood gun violence exposure and adult mental health outcomes varies by race/ethnicity and gender. These results highlight the need for gun violence preventions and interventions tailored to specific demographic groups to address the long-term mental health consequences of childhood gun violence exposure.

**Supplementary Information:**

The online version contains supplementary material available at 10.1007/s11524-025-01034-2.

## Introduction

Childhood exposure to firearm violence is a widespread and urgent public health problem in the United States—one that disproportionately impacts urban, racially marginalized communities [1]. Despite increasing recognition of the harmful effects of exposure to firearm violence, there is limited understanding of its long-term mental health associations, especially how these relationships vary by race/ethnicity and gender. This study addresses this critical gap by using national longitudinal data to examine: (1) the prevalence of childhood firearm exposure, (2) its associations with depressive symptoms, heavy episodic drinking, and daily cigarette smoking, and (3) how these associations differ across race/ethnicity and gender.

Firearm injury has become the leading cause of death for U.S. children and teenagers [2, 3]. However, deaths resulting from firearm assault only account for a fraction of the overall impact of gun violence. Firearm injury impacts children through direct exposure (e.g., being killed, injured, or threatened by a gun) and indirect exposure (e.g., witnessing gun violence, hearing gunshots, losing a friend or family member due to firearm injury) [4].

Indirect exposure to gun violence, such as hearing gunshots or witnessing gunfire, is widespread. Finklehor and colleagues estimated that approximately 13% of adolescents aged 14–17 in a nationally representative sample reported either hearing gunshots or witnessing someone being shot at some point in their lives [5]. Mitchell and colleagues reported that among a nonrepresentative sample of youth, ages 2 to 17, 41% reported ever seeing or hearing gun violence and 32% had such an experience within the past year. Additionally, 12.5% reported at least one direct victimization with a weapon, and 13.1% at least one indirect (or witnessed) victimization with a weapon [6].

These forms of exposure are disproportionately concentrated in structurally marginalized, urban areas, where gun violence is both a symptom and consequence of systemic disinvestment [7]. Black and Latinx people are disproportionately exposed to gun violence in the U.S [1, 8]. For example, scholars have found that Black and Latinx youth were 3–7 times more likely to be exposed to a gun homicide in the past year compared to white youth, with incidents typically occurring closer to home and more recently [1, 9]. Findings from a study analyzing child firearm injuries during the COVID-19 pandemic in four major U.S. cities indicated that Black children were 100 times, and Hispanic children 25 times, more likely than white children to be shot [10]. In addition to racial/ethnic and gender disparities in gun violence exposure persist through the life course [11, 12]. For example, findings from a longitudinal cohort study in Chicago showed that men are more likely to have been shot and have witnessed a person being shot compared to their women counterparts from childhood through mid-adulthood [9].

While exposure to gun violence, particularly in childhood, is a national public health concern, the long-term mental health consequences of childhood exposure to gun violence and variation in these consequences by race/ethnicity and gender are not well documented [13]. Existing research links exposure to firearm violence—both direct and indirect—to elevated risks of post-traumatic stress, depression, anxiety, suicidality, substance use, and quality of life [6, 13–17]. These psychological harms can persist into adulthood, affecting long-term well-being and functioning [18, 19]. For example, nonfatal shooting survivors and their family members report lasting trauma-related symptoms [20]. Even proximity to gun violence can impact child mental health: a Philadelphia-based study found that children living within a 4–5 block radius of a firearm incident were significantly more likely to visit emergency departments for acute mental health symptoms [21]. Recent evidence also highlight racial disparities in exposure and its mental health consequences, including elevated suicide ideation and behavior among Black adults [8]. However, most research to date has focused on short-term outcomes (e.g., in the following months after exposure), with limited attention to how these effects unfold over time or vary by race/ethnicity and gender. In addition, these studies have primarily used data drawn from smaller geographic areas such as cities or regions and used retrospective cohorts or cross-sectional studies.

In this study, we use national longitudinal data to examine how childhood exposure to firearm violence is associated with depressive symptoms and substance use behaviors in adulthood, and how these relationships differ by race/ethnicity and gender. In doing so, this research contributes to urban health scholarship by documenting the enduring psychological costs of gun violence, revealing patterns of racialized and gendered inequity, and identifying opportunities for trauma-informed, place-based intervention.

## Methods

### Data and Sample

This study used data drawn from the National Longitudinal Survey of Youth (NLSY) 1997–2019. The survey included approximately 9,000 youth who were between the ages of 12 and 16 in 1997, with an oversampling of Hispanic and non-Hispanic Black Americans. The total sample consisted of 8,984 individuals at the baseline. The NLSY97 survey was sponsored and directed by the U.S. Bureau of Labor Statistics and managed by the Center for Human Resource Research at The Ohio State University. Interviews were conducted by the National Opinion Research Center at the University of Chicago [22]. Depressive symptoms were assessed only in 2019 and 2021 in the NLSY data. Daily cigarette smoking was measured at every wave through 2015, and heavy episodic drinking was assessed at every wave through 2015 except 2013. Additional details on these outcome measures and their prevalence are provided in Supplemental Tables S1and S2. Because NLSY measures different outcomes at different waves, we used all the available data for each outcome. The final analytical samples included 7,314 individuals (13,530 person-years) for depressive symptoms, 8,960 individuals (107,182 person-years) for heavy episodic drinking, and 8,979 individuals (125,699 person-years) for daily cigarette smoking.

### Measurements

Race/ethnicity was based on participant self-report at the baseline. Four racial/ethnic groups were analyzed in the whole sample analyses: non-Hispanic White, non-Hispanic Black, Hispanic, and other/multiracial non-Hispanic. In the subgroup analyses, three racial/ethnic groups were examined: non-Hispanic White, non-Hispanic Black, and Hispanic. Participants' gender (male/female) was self-reported at the baseline.

#### Childhood Firearm Violence Exposure

In this study, we examined childhood exposure to firearm violence, categorized as either direct or indirect, before the age of 18. The NLSY97 initially inquired if participants, before turning 12, had witnessed someone being shot or shot at baseline in 1997. Follow-up questions during 1999 and in all subsequent waves until 2003 asked if participants, between ages 12 and 18, had been shot at or witnessed such incidents. This question was also asked in the first interview post-turning 18. We developed a cumulative dichotomous measure of exposure: participants were classified as 'exposed' if they affirmed witnessing or being a target of gunshot at least once before age 18, and 'not exposed' if they never reported such experiences.

#### Depressive Symptoms

Depressive symptoms were evaluated using the 7-item Center for Epidemiologic Studies Depression Short-Form (CES-D-SF) scale in 2019 and 2021 when respondents were ages 34–40, which has been validated against the larger 20-item CES-D scale designed to identify clinically significant depressive symptoms [23]. Participants were asked to rate their level of agreement, on a scale of 0 to 3, Scores ranged from 0 to 21, with higher scores indicating more pronounced depressive symptoms. We defined high levels of depressive symptoms as a binary variable, using a cut-off score of ≥ 8 on the CES-D-SF for our analyses [23].

#### Substance use

Substance use was assessed through self-reported information on heavy episodic drinking and daily cigarette smoking. We used data from wave 1 through wave 19 (1997–2015). Participants were asked about their heavy episodic drinking and daily cigarette smoking behaviors in each wave from baseline until wave 19 (2015). However, heavy episodic drinking was not measured in wave 18 (2013). Heavy episodic drinking was determined by the number of times respondents reported consuming five or more alcoholic beverages on a single occasion within the previous 30 days. Participants who indicated that they had consumed five or more beverages at least once were classified as engaging in heavy drinking [24]. Daily smoking was measured by asking participants about the number of days in the past month they smoked cigarettes. Respondents who reported smoking at least one cigarette daily during the past 30 days were categorized as daily smokers, while all others were classified as non-smokers/occasional smokers [24]. Details on outcome variables, including measurement levels, ages of participants at measurement, validity, and prevalence by race and gender subgroups, are provided in Supplemental Tables S1, S2, and S3.

#### Control Variables

We adjusted for factors that could associate with both exposure to gun violence and mental health or substance use outcomes. Socioeconomic variables included household income (inflation-adjusted to 2015 USD) and parental education (years of schooling of the most educated parent). Demographic variables—census region (northeast, north central/midwest, south, and west), urban/rural residence, and household size—capture place-based context and living arrangements. Age at baseline (12–16 years) accounts for developmental stage. Peer influence on smoking and drinking through youths’ perceived percentage of peers engaging in these behaviors (< 10% to 90% in five categories), served as a proxy for neighborhood and social-norm effects. These variables were measured at the baseline in 1997. For substance use outcomes, we also adjusted for time-varying age and survey year. These control variables have been previously justified in health research using the same dataset [24]. We did not include time-varying covariates of household socioeconomic position (e.g., income) because exposure to gun violence was measured as occurring anytime before age 18, and household socioeconomic position may act as a potential mediator between exposure to gun violence and mental health outcomes in early to mid-adulthood.

### Analytic Methods

Hierarchical generalized linear models (HGLM) [25] were used due to the hierarchical structure of the longitudinal data, where observations were observed multiple times, to control for clustering effects. Two-level logistic regressions with random intercepts for each person were performed. Multiple imputation by chained equations were used to impute missing predictor variables from the NLSY97. Analyses were replicated across the 30 datasets for the depressive symptoms outcome and 50 datasets for the heavy drinking and daily smoking outcomes and combined using Rubin’s rules [26]. We applied weights to account for the complex sampling design and respondent attrition.

## Results

At baseline (Table 1), the average adolescent was aged 14 years, living in a 4-person household. Slightly more than half of the adolescents sampled were men (51.3%). About two-thirds (66.5%) of the sample was non-Hispanic White, 15.4% was Black, and 12.86% was Hispanic. Most of the respondents resided in urban areas (72.5%), and more than a third (34.2%) of respondents reported living in the south. The mean household income was 77,200 in 2015 US dollars. The mean parental education was 13.7 years.

**Table 1:** Weighted descriptive statistics at the baseline

	**Whole Sample (n = 8,984)**	**Non-Hispanic White Sample (n = 4,406)**	**Non-Hispanic Black Sample (n = 2,335)**	**Hispanic Sample (n = 1,901)**
**Categorical variable**	**Weighted %**			
Race/ethnicity				
Non-Hispanic White	66.54			
Non-Hispanic Black	15.41			
Hispanic	12.86			
Other or multiracial non-Hispanic	5.2			
Gender				
Male	51.32	51.07	50.98	53.61
Female	48.68	48.93	49.02	46.39
Region				
Northeast	18.45	20.5	13.41	15.99
North central/Midwest	26.34	31.55	18.29	11.19
South	34.22	30.47	61.39	27.81
West	20.99	17.48	6.91	45
Geographic area				
Rural	27.54	34.43	18.36	9.41
Urban	72.46	65.57	81.64	90.59
**Continuous variable**	**Mean (SD)**			
Income (10 k)	7.72 (6.59)	8.76 (6.91)	4.59 (4.21)	5.14 (4.74)
Parental education (in years) †	13.73 (2.89)	14.28 (2.59)	12.94 (2.15)	11.60 (3.68)
Age	14.36 (1.50)	14.36 (1.50)	14.37 (1.50)	14.40 (1.50)
Household size	4.45 (1.44)	4.33 (1.29)	4.54 (1.78)	4.87 (1.63)

Table 2 shows weighted descriptive statistics of gun violence exposure in the full sample and by race/ethnicity and gender. About 16.2% of the respondents reported that they have been exposed to gun violence before the age of 18. The exposure to gun violence varies significantly by both race/ethnicity and gender. Non-Hispanic Black individuals reported the highest exposure at 32.4%, followed by Hispanic individuals at 19.8%, and non-Hispanic White individuals at 11.8%. Men experienced higher rates of gun violence exposure (20.6%) compared to women (11.4%). Among all groups, Black men had the highest prevalence of exposure, with 42.7% reporting gun violence during childhood.

**Table 2:** Childhood gun violence exposure by race/ethnicity and gender

	Total		Men		Women	
	Unweighted N	Weighted percentage	Unweighted N	Weighted percentage	Unweighted N	Weighted percentage
Whole Sample	1,746	16.16%	1,145	20.64%	601	11.43%
Non-Hispanic White Sample	517	11.84%	332	14.76%	185	8.80%
Non-Hispanic Black Sample	783	32.42%	517	42.74%	266	21.69%
Hispanic Sample	397	19.82%	268	25.90%	129	12.80%

Exposure to gun violence was significantly associated with increased risk of high levels of depressive symptoms, heavy episodic drinking, and daily cigarette smoking in early and middle adulthood. Table 3 displays results on the whole sample and subgroup analyses by race/ethnicity and gender. Childhood exposure to firearm violence was associated with greater odds (OR = 2.35, 95% CI = 1.80, 3.07) of experiencing high levels of depressive symptoms compared to those not exposed. We found racial/ethnic differences in these associations—gun violence exposure was significantly associated with experiencing high levels of depressive symptoms among non-Hispanic White (OR = 2.86, 95% CI = 1.91, 4.28) and Hispanic (OR = 1.98, 95% CI = 1.18, 3.33) sample, Among non-Hispanic Black participants, the association was positive but did not reach statistical significance at 0.05 level (OR = 1.46, 95% CI 0.99, 2.17).

**Table 3:** Odds Ratios of Gun Violence Exposure Associated with Depressive Symptoms, Heavy Episodic Drinking, and Daily Cigarette Smoking, Whole Sample and Differences by Race/Ethnicity and Gender

Variables	Whole Sample	Men Sample	Women Sample	Non-Hispanic White Sample	Non-Hispanic Black Sample	Hispanic Sample
	OR (95% CI)	OR (95% CI)	OR (95% CI)	OR (95% CI)	OR (95% CI)	OR (95% CI)
Depressive Symptoms ^a^						
Exposed to gun violence (ref: No)						
Yes	2.35***	1.71**	3.46***	2.86***	1.46*	1.98**
	(1.80 −3.07)	(1.17 −2.50)	(2.32 −5.17)	(1.91 −4.28)	(0.99 −2.17)	(1.18 −3.33)
Observations (person-year)	13,530	6,631	6,899	6,506	3,705	2,856
Respondents (person)	7,314	3,630	3,684	3,510	1,993	1,559
Heavy Episodic Drinking ^b^						
Exposed to gun violence (ref: No)						
Yes	1.40***	1.37***	1.41***	1.33***	1.55***	1.38***
	(1.24—1.57)	(1.18—1.59)	(1.17—1.70)	(1.12—1.58)	(1.28—1.87)	(1.10—1.74)
Observations (Person year)	107,182	54,695	52,487	51,972	28,355	22,845
Respondents (person)	8,960	4,586	4,374	4,390	2,332	1,896
Daily Cigarette Smoking ^c^						
Exposed to gun violence (ref: No)						
Yes	5.38***	5.13***	5.24***	8.22***	2.62***	2.38***
	(4.60—6.29)	(4.20—6.25)	(4.04—6.80)	(6.43—10.50)	(2.07—3.32)	(1.74—3.26)
Observations (Person-year)	125,699	63,414	62,285	61,313	32,920	26,772
Respondents (person)	8,979	4,595	4,384	4,402	2,335	1,900

Results were weighted. *** *p* < 0.01, ** *p* < 0.05, * *p* < 0.1

^a^: Results were based on 30 imputed datasets after applying Rubin’s rule. Model controlled for race/ethnicity, gender (when applicable), income, parental education, region, geographic location, household size, age all measured at the baseline in 1997), and survey year (time-varying)

^b, c^: Results were based on 50 imputed datasets after applying Rubin’s rule. Model controlled for time-invariant (e.g., race/ethnicity, gender (when applicable), income, parental education, region, geographic location, household size, and peer influence; all measured at baseline in 1997), and time-varying (age, age squared, and survey year) covariates

Gun violence exposure was significantly associated with higher odds of heavy episodic drinking in the whole sample analyses (OR = 1.40, 95% CI = 1.24, 1.57). Furthermore, gun violence exposure was associated with higher odds of heavy episodic drinking among all the racial/ethnic subsamples (OR NH White = 1.33, 95% CI = 1.12, 1.58); (OR NH Black = 1.55, 95% CI = 1.28, 1.87); (OR Hispanic = 1.38, 95% CI = 1.10, 1.74).

Participants who were exposed to gun violence before age 18 had five times higher odds of reporting daily cigarettes smoking (OR = 5.38, 95% CI = 4.60, 6.29) in the whole sample analyses. Significant racial/ethnic differences were found for daily cigarettes smoking. Gun violence exposure was associated with more than eight times higher odds of daily smoking among non-Hispanic White sample (OR = 8.22, 95% CI = 6.43, 10.50), whereas the odds ratios were lower for Black (OR = 2.62, 95% CI = 2.07, 3.32) and Hispanic respondents (OR = 2.38, 95% CI = 1.74, 3.26).

Racial/ethnic and gender disaggregated analyses results are represented in Table 4. Results indicate that childhood gun violence exposure was significantly associated with increased risk of high levels of depressive symptoms among non-Hispanic White men (OR = 1.82, 95% CI = 1.02, 3.27) and women (OR = 5.39, 95% CI = 2.79, 10.42) and Hispanic women (OR = 3.31, 95% CI = 1.62, 6.80). No significant relationships were found between gun violence exposure and CES-D among non-Hispanic Black men and women, and Hispanic men. Regarding heavy episodic drinking, exposure to gun violence was a significant predictor for non-Hispanic White women (OR = 1.46, 95% CI = 1.10, 1.94), non-Hispanic Black men (OR = 1.83, 95% CI = 1.42, 2.35), and Hispanic women (OR = 1.54, 95% CI = 1.06, 2.23). We did not find a significant association between gun violence exposure and heavy episodic drinking for non-Hispanic White men, non-Hispanic Black women, and Hispanic men. As for daily cigarette smoking, gun violence exposure was significantly associated with all subgroups. Notably, non-Hispanic White women who were exposed to gun violence were nine times higher odds of reporting smoking daily (OR = 9.35, 95% CI = 6.21, 14.09), compared to those who did not have such exposure.

**Table 4:** Odds Ratios of Gun Violence Exposure Associated with Depressive Symptoms, Heavy Episodic Drinking, and Daily Cigarette Smoking, Disaggregated Analyses by Race/Ethnicity and Gender

Variables	Non-Hispanic White men	Non-Hispanic White women	Non-Hispanic Black men	Non-Hispanic Black women	Hispanic men	Hispanic women
	OR (95% CI)	OR (95% CI)	OR (95% CI)	OR (95% CI)	OR (95% CI)	OR (95% CI)
Depressive Symptoms						
Exposed to gun violence (ref: No)						
Yes	1.82**	5.39***	1.33	1.62*	1.30	3.31***
	(1.02–3.27)	(2.79–10.42)	(0.75–2.36)	(0.95—2.75)	(0.60–2.81)	(1.62–6.80)
Observations (person-year)	3,282	3,224	1,758	1,947	1,367	1,489
Respondents (person)	1,785	1,725	956	1,037	764	795
Heavy Episodic Drinking						
Exposed to gun violence (ref: No)						
Yes	1.24*	1.46***	1.83***	1.24	1.34*	1.54**
	(0.99—1.54)	(1.10—1.94)	(1.42—2.35)	(0.93—1.66)	(1.00—1.789)	(1.06—2.23)
Observations (person-year)	27,266	24,706	13,761	14,594	11,625	11,220
Respondents (person)	2,274	2,116	1,168	1,164	975	921
Daily Cigarette Smoking						
Exposed to gun violence (ref: No)						
Yes	7.55***	9.35***	3.15***	1.90***	2.49***	2.23***
	(5.57—10.24)	(6.21—14.09)	(2.27—4.38)	(1.34—2.71)	(1.68—3.67)	(1.27—3.90)
Observations (person year)	31,550	29,763	16,034	16,886	13,480	13,292
Respondents (person)	2,281	2,121	1,169	1,166	976	924

Results were weighted. *** *p* < 0.01, ** *p* < 0.05, * *p* < 0.1

^a^: Results were based on 30 imputed datasets after applying Rubin’s rule. Model controlled for race/ethnicity, gender (when applicable), income, parental education, region, geographic location, household size, age (all measured at the baseline in 1997), and survey year

^b, c^: Results were based on 50 imputed datasets after applying Rubin’s rule. Model controlled for time-invariant (e.g., race/ethnicity, gender (when applicable), income, parental education, region, geographic location, household size, and peer influence; all measured at baseline in 1997), and time-varying (age, age squared, and survey year) covariates

We conducted additional analyses of two-way (gun violence × race/ethnicity; gun violence × gender) and three-way (gun violence × race/ethnicity × gender) interactions (Table S4). Wald tests results show that the race/ethnicity × gun violence interaction was significant for daily cigarette smoking, *F*(3) = 25.11, *p* < 0.001. Gender significantly moderated the association between gun violence and depressive symptoms *F*(1) = 8.29, *p* = 0.004.

## Discussion

This study examined how childhood exposure to gun violence is associated with mental health outcomes into mid-adulthood, providing unique contributions to the literature as data were drawn from a longitudinal, nationally representative sample. While previous studies have largely focused on shorter-term impacts or cross-sectional assessments, our findings demonstrate the enduring mental health outcomes associated with early firearm exposure, which could simultaneously fuel long-term health outcomes as well as behavioral health inequities. By disaggregating results by race/ethnicity and gender, we provided new insight into how structural inequities shape long-term outcomes in different populations. These findings not only confirm prior research linking violence exposure to psychological distress but also extend this work by highlighting persistent disparities across the life course. Our results underscore the need for trauma-informed interventions and culturally responsive policies aimed at supporting youth exposed to violence—especially in communities that have long faced systemic disinvestment and elevated exposure to gun violence. Furthermore, communities that experience the greatest exposure to gun violence lack access to mental health services, especially community level behavioral health providers as opposed to emergency departments [7].

More than 16% of the sample reported that they have been shot at or witnessed someone getting shot/shot at before the age of 18. Considering that the data from this study were drawn from a nationally representative sample, the findings from this study indicate that there is a high level of gun violence exposure among this cohort (born between 1980 to 1984). To contextualize, a study based in Chicago reported a higher exposure rate, with nearly 40% of individuals from a similar cohort experiencing gun violence by age 20, particularly during the violence peak in the 1990s. [9] We found that men had higher prevalence of exposure to firearm violence compared to women across all racial/ethnic groups. Furthermore, exposure to firearm violence during childhood was disproportionately higher for Black respondents (32.4%, 173.8% higher) and Hispanic respondents (19.8%, 67.4% higher) compared to their White counterparts (11.8%). Consistent with findings from previous research that found higher exposure to violence among Black youth [27], we found that young adult Black men had the highest level of exposure to firearm violence. Other researchers have documented that these disparities grew during the COVID-19 pandemic—Black children experienced the highest levels of gun violence exposure and the largest increase in exposure during the pandemic [28].

Exposure to gun violence during childhood is linked to poorer mental health and increased engagement in substance use behaviors during mid-adulthood in the whole sample analyses. Those who reported exposure to firearm violence before age 18 reported higher levels of depressive symptoms, heavy episodic drinking, and daily cigarettes smoking. Considering these findings, behavioral health agencies, schools, healthcare systems, and community-based providers should work collaboratively to ensure equitable access to culturally responsive and trauma-informed care for youth from structurally marginalized communities affected by violence. This includes proactive engagement with youth through advisory boards and other participatory strategies to ensure services are relevant and equity-centered. Given the elevated rates of alcohol and cigarette use among victims of gun violence exposure, substance use prevention and early intervention should be integrated into both post-trauma care and community-based programs. Finally, there is an urgent need for public policies that prioritize the prevention of firearm violence nationwide.

Our study findings indicate that the relationship between race/ethnicity, gender, childhood exposure to gun violence, and mental health outcomes varied depending on the outcome. Although childhood exposure to firearm violence was not significantly associated with higher levels of depressive symptoms among the Black sample, it is important to consider that lower rates of reported depression may be influenced by various factors. These could include measurement issues, variations in how depressive symptoms are experienced and expressed, differences in coping mechanisms, and disparities in access to mental health services [29–31]. Although gun violence exposure was significantly associated with daily cigarettes smoking across all racial/ethnic and gender subgroups, we found a more pronounced association among White sample, especially White women. Heavy episodic drinking was correlated with gun violence exposure among Black men, as well as White and Hispanic women. Exposure to gun violence carries divergent social implications for Black and Latinx communities compared to white Americans. Such differences suggest the need for further investigation into the racialization of gun violence exposure and how contextual factors affect its mental health consequences.

Results from prior research indicates that high levels of violence exposure, or exposure across various contexts, may lead to emotional desensitization, potentially concealing trauma symptoms, particularly in groups disproportionately impacted by such violence [32]. Our results highlight the importance of recognizing that gun violence exposure can elicit varied trauma responses across different communities. That is, differential exposure to gun violence across communities may result in distinct trauma manifestations. These findings suggest the need to expand our understanding of mental health consequences of gun violence exposure and underscore the significance of developing culturally tailored clinical and community interventions.

Overall, there is a dearth of research that has investigated the long-term mental health effects of gun violence exposure among minoritized communities suggests the need for continued research to further investigate the explanations and mechanisms of racialized and gendered experiences of trauma associated with gun violence exposure [33, 34]. The striking racial/ethnic inequities in exposure to gun violence highlighted in this study also reflect well-documented structural factors that disproportionately disadvantage communities of color. While our study does not directly measure structural racism, there are a preponderance of findings drawn from previous studies that indicate the effects of historical and contemporary policies and practices such as redlining, residential steering, and inequitable sentencing guidelines that undergird the foundation of inequities in firearm exposure [35]. Our study findings emphasize the necessity of equity-centered prevention strategies that take into account the effects of gender, race/ethnicity, and context among adolescents who were victimized by or witnessed firearm violence during childhood. Addressing these specific needs will be crucial in effectively mitigating the long-term impact of firearm violence on marginalized populations.

The rise in firearm violence during the COVID-19 pandemic is anticipated to have long-lasting effects, as it significantly heightened both indirect and direct exposure among young people in the United States. Consequently, it is vital to intensify the implementation of public health initiatives aimed at curbing this surge in violence and addressing its aftermath. This study provides evidence to inform racial/ethnic tailored community-based outreach trauma-informed programing and services to effectively address the situation in the decades to come.

## Limitations

This study has several limitations that should be noted. Foremost, we could not conduct subgroup analyses for other racial/ethnic groups, such as Native Americans, Asian Americans, as well as Native Hawaiians and Pacific Islanders. The small number of participants from these groups precluded a detailed examination. Similarly, the data did not provide sufficient information to differentiate participants within Hispanic sample based on nativity or nationality. Second, there are limitations of measurements of childhood gun violence exposure. Although the NLSY97 is prospective, it relied on participants accurately recalling and reporting all information related to exposure to firearm violence, introducing the possibility of recall bias. Our study combined direct and indirect exposure to firearm violence and did not distinguish between the severity of the violence exposed. Therefore, we did not capture the potentially different impacts of specific types and severity of forearm violence exposure. For example, the mental health consequence of chronic exposure to violence is different from experiencing a homicide [15]. Additionally, whereas this study focuses on gun violence exposure, it is important to note that individuals exposed to firearm violence are also likely to experience other forms of violence (e.g., stabbings, assaults).^14^ Our analysis does not disentangle these potential overlapping exposures, which may confound the associations observed. Furthermore, we used self-reported mental health outcome measures. Future studies should consider using clinical diagnostic measures of mental health.

Finally, the relationships between childhood gun violence exposure and mental health and substance use outcomes were associational instead of causal. We did not account for other confounding factors, such as neighborhood segregation. Furthermore, it is important to note that exposure to violence can be influenced by and co-occur with structural factors, including inadequate access to healthcare, housing, and education, as well as police brutality. These structural factors can contribute to psychological distress, which was not considered in this study.

## Future Research

This study shows racialized and gendered associations between gun violence exposure and mental health outcomes. Additional research is warranted to elucidate the complex mechanisms of exposure to firearm violence on various aspects of mental and behavioral health across race/ethnicity and gender. Future research should (1) examine both short-term and long-term mental health consequences (including suicide ideation and attempt) of exposure to firearm violence, and (2) seek to understand the mechanism of how different sociodemographic groups address mental health consequence of firearm violence exposure through qualitative and mixed-method approaches, and (3) continue to understand the variations in the link between firearm violence exposure and health by race/ethnicity and gender that were identified in this study.

## Conclusions

This study highlights the diverse connections between firearm violence exposure and mental health, and these connections vary across different racial/ethnic and gender groups. Understanding these variations is crucial in addressing the root causes of health disparities in populations disproportionately affected by gun violence. Taken together, findings underline the importance of adopting both comprehensive and equity-centered interventions to address exposure to firearm violence effectively in the U.S.

## Supplementary Information

Below is the link to the electronic supplementary material.

Supplementary file1 (DOCX 27.5 KB)
