# Supplementary material for: Examining Long-Term Mental Health Outcomes Associated with Childhood Gun Violence Exposure: Variations by Race/Ethnicity and Gender
Source: J Urban Health. 2025 Dec 6;102(6):1141–51. doi: 10.1007/s11524-025-01034-2 (PMC12738394; doi:10.1007/s11524-025-01034-2)
Supplement: Supplementary file 1 — Supplementary file1 (DOCX 27.5 KB) [file 11524_2025_1034_MOESM1_ESM.docx]

Supplemental materials

Table S1. Mental health outcome measures

| **Outcomes** | **Description** | **Response options** | **Data Management** | **Years available** | **Citation** |
| --- | --- | --- | --- | --- | --- |
| **Depressive symptoms** | Center for Epidemiologic Studies Depression Short-Form (CES-D-SF) 7 items. Please tell me how often you felt this way during the past week. Statements are as follows: 1. "I did not feel like eating, my appetite was poor", 2. "I had trouble keeping my mind on what I was doing"; 3. "I felt depressed"; 4. "I felt that everything was an effort"; 5. "My sleep was restless"; 6. "I felt sad"; 7. "I could not get “going” | Participants rate how often they experienced symptoms in the past week using a scale ranging from 1). 0 (rarely/none of the time/1 day); to 3). (most /all of the time/5-7 days). | Scores ranged from 0 to 21 with higher scores indicating higher levels of depressive symptoms. Cut off at ≥8 is higher levels of depressive symptoms=1, otherwise =0. | 2019, 2021 | ^1,2^ |
| **Heavy episodic drinking** | Respondents were asked "On how many days did you have five or more drinks on the same occasion during  the past 30 days? By occasion we mean at the same time or within hours of each  other." | Participants were given the following options: 1). 0; 2). 1-4; 3). 5 to 9; 4). 10 to 14; 5). 15 to 19; 6). 20 to 24; 7). 25 to 29; 8). 30 | For each wave, those who indicated they had done so on at least one occasion were coded as having heavy drinking behavior=1, otherwise =0. | 1997, 1998, 1999, 2000, 2001, 2002, 2003, 2004, 2005, 2006, 2007, 2008, 2009, 2010, 2011, 2015 | ^3^ |
| **Daily cigarette smoking** | Respondents were asked "During the past 30 days, on how many days did you smoke a cigarette?" | Participants were given the following options: 1. 0; 2. 1-4; 3. 5 to 9; 4. 10 to 14; 5. 15 to 19; 6. 20 to 24; 7. 25 to 29; 8. 30 | For each wave, those who answer 30 were coded as daily smokers=1, otherwise were coded as nonsmokers/occasional smokers =0 | 1997, 1998, 1999, 2000, 2001, 2002, 2003, 2004, 2005, 2006, 2007, 2008, 2009, 2010, 2011, 2013, 2015 | ^4,5^ |

References

1. Levine SZ. Evaluating the seven-item Center for Epidemiologic Studies Depression Scale short-form: a longitudinal US community study. *Soc Psychiatry Psychiatr Epidemiol*. 2013;48(9):1519-1526. doi:10.1007/s00127-012-0650-2

2. Hawkinson CB, Andrea SB, Hajat A, et al. A cross-sectional analysis of work schedule notice and depressive symptoms in the United States. *SSM - Population Health*. 2023;22:101413. doi:10.1016/j.ssmph.2023.101413

3. Sun S. Racial/Ethnic Heterogeneity in Parental Wealth and Substance Use from Adolescence to Young Adulthood. *J Racial and Ethnic Health Disparities*. Published online December 19, 2023. doi:10.1007/s40615-023-01893-y

4. Epperson AE, Gonzalez M, Skorek M, Song AV. Challenging Assumptions About Race/Ethnicity, Socioeconomic Status, and Cigarette Smoking Among Adolescents. *J Racial and Ethnic Health Disparities*. Published online February 4, 2021. doi:10.1007/s40615-021-00974-0

5. Wang TW, Gentzke AS, Creamer MR, et al. Tobacco product use and associated factors among middle and high school students—United States, 2019. *MMWR Surveillance Summaries*. 2019;68(12):1.

Table S2. Weighted descriptive statistics of the outcome variables, by year

| Year | Respondents age range at time of survey | Higher levels of depressive symptoms (%) | Heavy Episodic Drinking (%) | Daily Cigarettes Smoking (%) |
| --- | --- | --- | --- | --- |
| 1997 | 12-18 | NA | 14.05 | 6.92 |
| 1998 | 13-19 | NA | 22.14 | 12.85 |
| 1999 | 14-20 | NA | 29.17 | 15.69 |
| 2000 | 15-21 | NA | 33.48 | 18.85 |
| 2001 | 16-22 | NA | 38.17 | 21.05 |
| 2002 | 17-23 | NA | 41.21 | 22.79 |
| 2003 | 18-24 | NA | 43.87 | 23.7 |
| 2004 | 19-25 | NA | 44.22 | 24.24 |
| 2005 | 20-26 | NA | 45.43 | 24.07 |
| 2006 | 21-27 | NA | 44.59 | 24.49 |
| 2007 | 22-28 | NA | 43.49 | 23.53 |
| 2008 | 23-29 | NA | 42.81 | 23.26 |
| 2009 | 24-30 | NA | 40.18 | 22.8 |
| 2010 | 25-31 | NA | 37.58 | 21.84 |
| 2011 | 26-32 | NA | 37.82 | 21.85 |
| 2013 | 28-34 | NA | NA | 25.87 |
| 2015 | 30-36 | NA | 32.15 | 24.27 |
| 2019 | 34-40 | 11.79 | NA | NA |
| 2021 | 36-42 | 13.77 | NA | NA |

Table S3. Weighted descriptive statistics of the outcome variables, by race, ethnicity, and gender

|  | Higher levels of depressive symptoms (%) | Heavy Episodic Drinking (%) | Daily Cigarettes Smoking (%) |
| --- | --- | --- | --- |
| Whole sample | 12.96 | 36.80 | 35.84 |
| Men sample | 9.55 | 43.60 | 42.53 |
| Women sample | 16.32 | 29.58 | 28.94 |
| NH White sample | 13.21 | 41.40 | 40.09 |
| NH Black sample | 13.34 | 20.81 | 20.66 |
| Hispanic sample | 10.84 | 35.05 | 34.56 |
| NH White men sample | 9.52 | 48.11 | 46.83 |
| NH White women sample | 16.86 | 34.23 | 33.16 |
| NH Black men sample | 10.53 | 26.77 | 26.42 |
| NH Black women sample | 15.99 | 14.97 | 15.00 |
| Hispanic men sample | 7.47 | 42.60 | 41.86 |
| Hispanic women sample | 14.31 | 26.47 | 26.46 |

Table S4. Wald test of interaction effects

|  | Depressive symptoms | Heavy episodic drinking | Daily cigarette smoking |
| --- | --- | --- | --- |
| Gun violence * Race/ethnicity | F=1.24, df=3, p=0.29 | F=0.67,df=3, p=0.57 | **F=25.11, df=3, p=0.00** |
| Gun violence *Gender | **F=8.29, df=1, p=0.004** | F=0.01,df=1, p=0.93 | F=2.39, df=1, p=0.12 |
| Gun violence * Race/ethnicity*Gender | F=2.48, df=3, p=0.06 | F=1.15, df=3, p=0.33 | F=0.54, df=3, p=0.66 |

Note: Bold indicates p < 0.05; results weighted and pooled from 30 (depressive symptoms) or 50 (drinking, smoking) imputations using Rubin’s rule; models adjusted for socioeconomic and demographic fixed and random effects as shown in Table 3.
